# Integer division by constants: optimal bounds

**DOI:** 10.1016/j.heliyon.2021.e07442

**Published:** 2021-06-29

**Authors:** Daniel Lemire, Colin Bartlett, Owen Kaser

**Affiliations:** a Université du Québec (TELUQ), 5800 Saint-Denis, Montreal, Quebec, Canada; b Computer Science Department, UNB Saint John, New Brunswick, Canada

**Keywords:** Integer division, Compiler optimization, Tight bounds

## Abstract

The integer division of a numerator *n* by a divisor *d* gives a quotient *q* and a remainder *r*. Optimizing compilers accelerate software by replacing the division of *n* by *d* with the division of c⁎n (or c⁎n+c) by *m* for convenient integers *c* and *m* chosen so that they approximate the reciprocal: c/m≈1/d. Such techniques are especially advantageous when *m* is chosen to be a power of two and when *d* is a constant so that *c* and *m* can be precomputed. The literature contains many bounds on the distance between c/m and the divisor *d*. Some of these bounds are optimally tight, while others are not. We present optimally tight bounds for quotient and remainder computations.

## Highlights

• Generalizes theory on how to replace a division by a multiplication followed by a shift.
• Includes both the computation of the quotient and remainder.
• Introduces several new tighter bounds.

## 1 Introduction

The problem of computing the integer division given constant divisors has a long history in computer science [1], [2], [3], [4]. Granlund and Montgomery [5] present the first general-purpose algorithms to divide integers by constants using a multiplication and a division by a power of two: their work was adopted by the GNU Compiler Collection (GCC). Given any non-zero 32-bit divisor known at compile time, the optimizing compiler can replace the division by a multiplication followed by a shift. Warren [6] improved on the Granlund and Montgomery technique by deriving a better bound that gives a wider range of choices. Warren's better approach is found in LLVM's Clang compiler. Many optimizing compilers rely on equivalent techniques, either based on the original Granlund-Montgomery article or on Warren's technique.

Robison [7] describes a slightly superior alternative for some divisors in that we multiply and add the multiplier before dividing by a power of two (henceforth the multiply-add technique). Though it comes at the cost of an addition, it allows one to choose a smaller multiplier, which can be advantageous. Robison's approach is implemented in the popular libdivide library [8].

Most of the literature is focused on the computation of the quotient *q* of the division of *n* by *d*. From the quotient *q*, we can compute the remainder as n−q⁎d. We can also compute the remainder directly [9] without first computing the quotient: it is given by taking remainder of c⁎n divided by *m*, and then multiplying it by *m*. However, for the remainder and the quotient to be exact, it is necessary that c/m approximates 1/d more closely than if we merely need the quotient.

From the computation of remainders, we can derive a divisibility check, that is check whether *d* divides *n*, or, equivalently, check that *n* is a multiple of *d*. Though it may seem that computing the remainder and checking whether it is zero is efficient, we can simplify and accelerate the algorithm by avoiding the computation of the remainder.

The literature commonly assumes that *m* is a power of two. We approach the problem more generally, letting *m* and *c* be any integer, and restricting the numerator to an interval [0,N] where *N* can be any integer. It makes our exposition more general, while simplifying the notation.

Some our novel contributions are as follows:

• We improve Robison's bound [7], in a manner similar to how Warren improved Granlund and Montgomery's bound. That is, we provide an optimal bound for the multiply-add technique.^1^
• We derive a new tighter bounds for computing the quotient directly and checking the divisibility, thus improving on the work of Lemire at al. [9]
• We show that we can adapt Robison's technique to compute remainders directly and derive a novel bound. We adapt the multiply-add technique for the purpose of a divisibility check. To our knowledge, these results are novel.

All our bounds on how close c/m must be to 1/d are optimal and form necessary and sufficient conditions. Table 1 presents our core results in concise manner.

**Table 1:** Summary of main results. Throughout, all values are non-negative integers, the divisor is non-zero *d* > 0, and the numerator is bounded by *N* ≥ *d*, so that *n* ∈ [0,*N*]. We add the constraint that *c* ∈ [0,*m*) so that *c* is as small as possible.

Theorem 1 and Warren [11]	
statement:	division(*n*,*d*)=division(*c*⁎*n*,*m*) for all *n* ∈ [0,*N*]
condition:	$1/d\leq c/m<\left(1+\frac{1}{N-\mathrm{remainder}(N+1,d)}\right)1/d$

Theorem 2 (novel, improves Lemire et al. [9, Theorem 1])	
statement:	division(*n*,*d*)=division(*c*⁎*n*,*m*) and remainder(*n*,*d*)=division(remainder(*c*⁎*n*,*m*)⁎*d*,*m*) for all *n* ∈ [0,*N*]
condition:	$1/d\leq c/m<\left(1+\frac{1}{N}\right)1/d$

Proposition 1 (novel, generalizes Lemire et al. [9])	
statement:	*d* divides *n* ∈ [0,*N*] if and only if remainder(*c*⁎*n*,*m*)<*c*
condition:	$1/d\leq c/m<\left(1+\frac{1}{N}\right)1/d$

Theorem 3 (improves Robison [7])	
statement:	division(*n*,*d*)=division(*c*⁎*n* + *c*,*m*) for all *n* ∈ [0,*N*]
condition:	$\left(1-\frac{1}{N-\mathrm{remainder}(N,d)+1}\right)1/d\leq c/m<1/d$

Theorem 4 (novel)	
statement:	division(*n*,*d*)=division(*c*⁎*n* + *c*,*m*) and remainder(*n*,*d*)=division(remainder(*c*⁎*n* + *c*,*m*)⁎*d*,*m*) for all *n* ∈ [0,*N*]
condition:	$\left(1-\frac{1}{N+1}\right)1/d\leq c/m<1/d$

Proposition 2 (novel)	
statement:	*d* divides *n* ∈ [0,*N*] if and only if remainder(*c*⁎*n* + *c*,*m*)<*c*
condition:	$\left(1-\frac{1}{N+1}\right)1/d\leq c/m<1/d$

## 2 Other related work

The problem of quickly computing the division by a constant in computers dates back to at least the 1970s. Jacobsohn [2] shows that we can divide by an odd integer by multiplying by a fractional inverse, followed by some rounding. Artzy et al. [1] describe a related algorithm to divide multiples of a known divisor (exact division). Li [3] presents algorithms for integer division by all odd integers up to 55 [11, § 10-18]. Divisions are executed as series of “shift and add” instructions.

Magenheimer et al. [12] describe how to compute the division of integers by odd divisors as a multiplication and an addition followed by a division by a power of two. Their approach was later refined by Robison [7]. Similarly, Granlund and Montgomery's approach [5] (without an intermediate addition) was refined by Cavagnino and Werbrouck [13], and later by Warren [11]. As remarked by Robison [7], the two approaches (with and without an intermediate addition) are complementary: we can choose one or the other depending on the divisor. We review and elaborate on this complementarity in § 6.

To our knowledge, the latest work on the software acceleration of the division by constants was Lemire et al. [9]. They revisited two specific problems: the direct computation of the remainder—without first computing the quotient—and the related divisibility tests. Compared to optimizing compilers that compute the remainder by first computing the quotient, they found that their direct approach could be up to 30% faster. Their divisibility test could be twice as fast as the code produced by popular optimizing compilers and libraries. It can also be up to twice as fast as the state-of-the-art divisibility check proposed by Granlund and Montgomery [5]. They did not consider the multiply-add approach, a gap that we fill with § 5. We also make their main result [9, Theorem 1] tighter (see Theorem 2). We similarly improve mathematically on their divisibility check [9, Proposition 1] (see Proposition 1). Our improvements may not immediately result in improved software performance, but they fill a conceptual gap. The systematic computation of the remainder directly as proposed by Lemire et al., without first computing the quotient, has received attention in the hardware and circuit literature [14], [15], [16], [17] but had never been generally exploited in software as far as we know. One practical exception was the work by Vowels [4] who described the direct computation of both the quotient and remainder, in the special case where we divide by 10.

## 3 Technical preliminaries

For non-negative real numbers *z*, floor(z) is the greatest integer no larger than *z*. It is a monotonic function: if $z_{1}\geq z_{2}$ then $\mathrm{floor}(z_{1})\geq \mathrm{floor}(z_{2})$.

We define division(x,y)≡floor(x/y) and

(1) remainder(x,y)≡x−division(x,y)⁎y

(2) =x−floor(x/y)⁎y

for positive real numbers x,y with the constraint that y≠0. We have that remainder(x,y)∈[0,y). If *y* is an integer and *x* is not an integer, then remainder(x,y)≠0. By definition, we always have that x=division(x,y)⁎y+remainder(x,y).

Lemma 1 *Consider a positive integer* d>0*, a non-negative integer n and a non-negative real number x. We have that* remainder(n,d)=floor(remainder(x,d)) *and* division(n,d)=division(x,d) *if and only if* n≤x<n+1*.*

Proof (⇐) We can verify that if n≤x<n+1, the previous two conditions are satisfied.

(⇒) Assume that remainder(n,d)=floor(remainder(x,d)) and division(n,d)=division(x,d). We have

(3) remainder(n,d)≤remainder(x,d)

(4) <remainder(n,d)+1.

By expanding out remainder(n,d)=floor(remainder(x,d)), we have that

(5) n−floor(n/d)⁎d≤x−floor(x/d)⁎d

(6) <n−floor(n/d)⁎d+1.

Expanding out division(n,d)=division(x,d) and multiplying by *d*, we have floor(n/d)⁎d=floor(x/d)⁎d. We establish the lemma by adding this last equation to the previous inequality. □

## 4 Multiply-divide results

Given a non-negative numerator *n* and non-zero divisor *d*, we want to show that by choosing integer constants *c* and *m* carefully, we can compute division(n,d) and remainder(n,d) by starting from c⁎n and dividing by *m*.

### 4.1 Quotient

We want to find *c* and *m* such that division(n,d)=division(c⁎n,m). Intuitively, this equation implies that n/d≈c⁎n/m and n≈c⁎n⁎d/m. Let us formalize this intuition.

For any non-negative real number *x* and non-negative integer *Q*, we have that floor(x/d)=Q is equivalent to x∈[Qd,Qd+d). Letting Q=floor(n/d) and x=c⁎n⁎d/m, we get d⁎floor(n/d)≤c⁎n⁎d/m<d⁎floor(n/d)+d. Since d⁎floor(n/d)=n−remainder(n,d), floor(x/d)=division(c⁎n,m) and Q=division(n,d), we have that division(n,d)=division(c⁎n,m) is equivalent to n−remainder(n,d)≤c⁎n⁎d/m<n−remainder(n,d)+d.

Consider a range of integer numerators n∈[0,N] for some maximal integer numerator N≥d. We want this equation to hold for all *n*. The equation is satisfied trivially when n=0 and d>0. Suppose that n>0 and rewrite the inequalities as (n−remainder(n,d))/n≤c⁎d/m<(n−remainder(n,d)+d)/n.

Given any n∈[1,N] for some integer N≥d, we have that the leftmost expression (n−remainder(n,d))/n=1−remainder(n,d)/n is largest and equal to 1 when remainder(n,d)=0. Meanwhile the rightmost expression 1+(d−remainder(n,d))/n is smallest when *n* is as large as possible with remainder(n,d)=d−1. To prove this bound, partition the possible numerators into sets $N_{k}=\{n\in [0,N]\;|\;\mathrm{remainder}(n,d)=k\}$. Fixing *N* and *d*, we seek the value n∈[1,N] minimizing f(n)=1+(d−remainder(n,d))/n. For $n\in N_{k}$ we have f(n)=1+(d−k)/n which is minimized for the largest member of $N_{k}$. Let *v* be the largest member of $N_{d-1}$; we have f(v)=1+1/v. We see that the values of *n* in [N−d+1,N] are the minimizing values in each $N_{k}$. Among these, we can show *v* minimizes *f*.

• Consider any n∈[N−d+1,N] with n>v. Write it as n=v+k with k>0 and k≤d−1 and so remainder(n,d)=k−1 and f(n)=1+(d−k+1)/(v+k). As *k* increases, the numerator decreases and the denominator increases, we have that the minimum is reached when *k* is largest (d−1), in which case f(n)=1+2/(v+d−1)≥1+2/(v+v)=1+1/v=f(v), since v≥d−1.
• Consider any n∈[N−d+1,N] with n<v. Write *n* as v−d+k, so remainder(n,d) is again k−1. We have f(n)=1+(d−k+1)/(v−d+k). Again, as *k* increases, the numerator decreases and the denominator increases, we have that the minimum is reached when *k* is largest (d−1) in which case f(n)=1+2/(v−1)<1+1/v=f(v).

Thus n=v minimizes f(n). We have shown Lemma 2 because v=N−remainder(N+1,d).

Lemma 2 *Given an integer* d>0*, the value of* 1+(d−remainder(n,d))/n *over* n=0,1,…,N *is minimized when n is* n=N−remainder(N+1,d)*.*

Hence we have that $1\leq c⁎d/m<1+\frac{1}{N-\mathrm{remainder}(N+1,d)}$ is equivalent to division(n,d)=division(c⁎n,m) for all n∈[0,N].

Theorem 1 *Consider an integer divisor* d>0 *and a range of integer numerators* n∈[0,N] *where* N≥d *is an integer. We have that* division(n,d)=division(c⁎n,m) *for all integer numerators n in the range if and only if*

(7) $$1/d\leq c/m<\left(1+\frac{1}{N-\mathrm{remainder}(N+1,d)}\right)1/d.$$

Remark 1 Granlund and Montgomery [5] have an upper bound of c/m≤(1+1/(N+1))/d as a sufficient (but not necessary) condition. A bound equivalent to Theorem 1 is derived by Warren [6].

Once we have a pair of inequalities as in Theorem 1, we can solve for *c* and *m*. It is always possible to do so: we can verify that c=1, m=d is always a solution. However, we may have further constraints on *c* and *m*: maybe we require *m* to be a power of two. We can show that as long as we can choose *m* arbitrarily large, there is always a solution. Letting K=N−remainder(N+1,d), we can rewrite the inequalities as $m/d\leq c<\left(1+\frac{1}{K}\right)\frac{m}{d}$. Thus if *c* is to be as small as possible, we must have that c=ceiling(m/d). It remains to solve for *m* such that $\mathrm{ceiling}\;\left(\frac{m}{d}\right)<\left(1+\frac{1}{K}\right)\frac{m}{d}$. Because ceiling(m/d)−m/d<1, we have that the inequality is always satisfied when m≥K⁎d=(N−remainder(N+1,d))⁎d. This bound indicates that it is always possible to find a solution, by picking *m* large enough.

### 4.2 Remainder

From the quotient division(c⁎n,m), we get the quotient of the division of *n* by *d*; it is maybe intuitive that we can derive the remainder of the division of *n* by *d* from remainder(c⁎n,m).

Formally, we want to find integer constants c>0 and m>0 such that for any integer numerator n∈[0,N] and integer divisor d>0, we have that remainder(n,d)=division(remainder(c⁎n,m)⁎d,m).

If we find *c* and *m* such that remainder(n,d)=division(remainder(c⁎n,m)⁎d,m) is satisfied, then replacing *c* with c+m or c+2m would still work: in fact remainder(c⁎n,m)=remainder(c⁎n+k⁎m⁎n,m)=remainder((c+k⁎m)⁎n,m) for any integer *k*. Thus we require *c* to be in [0,m).

With this constraint (c∈[0,m)), we are able to show (see Lemma 3) that the ability to compute remainders via remainder(n,d)=division(remainder(c⁎n,m)⁎d,m) implies that the quotient of *n* divided by *d* is given by division(n,d)=division(c⁎n,m). Intuitively, it is strictly more difficult to compute the remainder than to compute the quotient. Hence, if we just need the remainder, and not the quotient, we cannot relax our conditions when c∈[0,m).

Lemma 3 *Consider an integer divisor* d>1*. Suppose that we have integer constants c and m such that* c∈[0,m) *and* remainder(n,d)=division(remainder(c⁎n,m)⁎d,m) *for all numerators* n∈[0,N] *then we must have that* division(n,d)=division(c⁎n,m)*.*

Proof When n=0, we have that division(n,d)=division(c⁎n,m) holds trivially. Since c∈[0,m) then c⁎(n+1)−c⁎n<m so when division(c⁎n,m) increases following an increment of *n* by one, it must increase by at most one. We just have to show that it happens exactly when remainder(n,d)=0.

We have c⁎n=remainder(c⁎n,m)+m⁎division(c⁎n,m). The left side of this equation increases by *c* exactly when *n* is incremented by one. When division(c⁎n,m) increases by one, then it contributes *m* to the right side. Since m>c, we have that an increase of division(c⁎n,m) corresponds to a decrease of remainder(c⁎n,m).

However, we have that remainder(n,d)=division(remainder(c⁎n,m)⁎d,m). From this equation, we have that whenever remainder(n,d) increases when we increment *n* by one, then remainder(c⁎n,m) must also increase. We know that when *n* is incremented, then either remainder(n,d) increases by one, or goes back to zero. It is not possible for remainder(n,d) to increase if remainder(c⁎n,m) decreases: it must therefore be that a decrease in remainder(c⁎n,m) corresponds to remainder(n,d)=0. Thus we have that an increase of division(c⁎n,m) following an increment of *n* corresponds remainder(n,d)=0. It follows that division(n,d)=division(c⁎n,m). □

We still must derive the conditions on *c* and *m*. We can expand the condition that remainder(n,d)=division(remainder(c⁎n,m)⁎d,m) as follows:

(8) $$\mathrm{remainder}(n,d)=\mathrm{floor}\;\left(\frac{\left(c⁎n-\mathrm{floor}\;\left(\frac{c⁎n}{m}\right)⁎m\right)⁎d}{m}\right)$$

(9) $$=\mathrm{floor}\;\left(c⁎n⁎d/m-\mathrm{floor}\;\left(\frac{c⁎n⁎d/m}{d}\right)⁎d\right)$$

(10) =floor(remainder(c⁎n⁎d/m,d)).

Then by Lemma 1, we have that the two constraints (remainder(n,d)=floor(remainder(c⁎n⁎d/m,d)) and division(n,d)=division(c⁎n⁎d/m,d)) are equivalent to n≤c⁎n⁎d/m<n+1, or m/d≤c<(1+1/n)m/d. This condition should hold for all applicable values of *n*, and thus we choose to use the maximal value of *n* (i.e., *N*) as it provides the tightest bound — so an equivalent expression is m/d≤c<(1+1/N)m/d.

We have derived the following theorem.

Theorem 2 *Consider an integer divisor* d>0 *and a range of integer numerators* n∈[0,N] *where* N≥d *is an integer. We have that* division(n,d)=division(c⁎n,m) *and* remainder(n,d)=division(remainder(c⁎n,m)⁎d,m) *for all integer numerators n in the range if and only if*

(11) $$1/d\leq c/m<\left(1+\frac{1}{N}\right)1/d.$$

We can check that the conditions of Theorem 2 are always met with c=ceiling(m/d) and m≥N⁎d.

Remark 2 In previous work [9, Theorem 1], Lemire et al. reported an upper bound of c/m≤(1+1/(N+1))1/d as a sufficient (but not necessary) condition. For the difference to matter, we need that there is an integer in the interval ((1+1/(N+1))m/d,(1+1/N)m/d). It happens in some instances, for example if N=10, d=5, and $m=2^{5}$, we have that 7∈((1+1/(N+1))m/d,(1+1/N)m/d). However, in the previous work [9], Lemire et al. considered only the case where m=N+1. We can show that if m=N+1 and m≥3 then the earlier bound is tight. Indeed, if there is an integer *z* in ((1+1/(N+1))m/d,(1+1/N)m/d) then there must be an integer z⁎d in ((1+1/(N+1))m,(1+1/N)m). Substituting N=m−1, the interval becomes (m+1,m+1+1/(m−1)): because m+1 is an integer and 1/(m−1)≤1/2, there is no integer in this interval. Hence, there cannot be an integer in ((1+1/(N+1))m/d,(1+1/N)m/d) and the earlier bound is tight.

If we only desire the remainder, and not the quotient, we can lift the restriction that c∈[0,m): we can replace *c* by c+k⁎m for any integer *k*.

### 4.3 Check for divisibility

We have that *n* is a multiple of *d* if and only if remainder(n,d)=0. Given Theorem 2, we can check whether remainder(n,d)=0 by checking whether division(remainder(c⁎n,m)⁎d,m)=0. In turn, we have that this last equation holds if and only if remainder(c⁎n,m)⁎d<m or remainder(c⁎n,m)<m/d. Thus remainder(c⁎n,m)<m/d is a divisibility test. However, we show the more elegant result that remainder(c⁎n,m)<c is a divisibility test (see Proposition 1).

By the assumption of Theorem 2, we have that m/d≤c. Thus if *n* is a multiple of *d*, then we have that remainder(c⁎n,m)<c. We need to prove the counterpart, that remainder(c⁎n,m)<c implies that *n* is a multiple of *d*. By Theorem 2, we have that division(n,d)=division(c⁎n,m). Hence we have that division(c⁎n,m)=division(c⁎(n−remainder(n,d)),m) since *n* and n−remainder(n,d) have the same quotient with respect to *d*. When two values $z_{1},z_{2}$ have the same quotient ($\mathrm{division}(z_{1},m)=\mathrm{division}(z_{2},m)$) then their difference must be captured by their remainders: $z_{2}-z_{1}=\mathrm{remainder}(z_{2},m)-\mathrm{remainder}(z_{1},m)$. In this case, taking $z_{1}=c⁎n$ and $z_{2}=c⁎(n-\mathrm{remainder}(n,d))$, we have that their difference is c⁎remainder(n,d). It follows that remainder(c⁎n,m)−remainder(c⁎(n−remainder(n,d)),m)=c⁎remainder(n,d) and therefore c⁎remainder(n,d)≤remainder(c⁎n,m). Thus if remainder(c⁎n,m)<c, we have c⁎remainder(n,d)<c which implies remainder(n,d)=0.

Proposition 1 *Consider an integer divisor* d>0*. We have that d divides* n∈[0,N] *if and only if* remainder(c⁎n,m)<c *subject to the condition that*

(12) $$1/d\leq c/m<\left(1+\frac{1}{N}\right)1/d.$$

Proposition 1 selects a value of *c* in [0,m) when d>1.

## 5 Multiply-add-divide results

Some authors [7], [12] have considered the case where we replace the division by a formula of the multiply-add from floor((c⁎n+b)/m) for some *b*. The benefit of the multiply-add approach is that it may allow one to pick a smaller value of *c*, compared to the simpler form floor(c⁎n/m). The derivations are nearly identical as in § 4, so we just give our results.

Theorem 3 *Consider an integer divisor* d>0 *and a range of integer numerators* n∈[0,N] *where* N≥d *is an integer. We have that* division(n,d)=division(c⁎n+c,m) *for all integer numerators n in the range if and only if*

(13) $$\left(1-\frac{1}{N-\mathrm{remainder}(N,d)+1}\right)1/d\leq c/m<1/d.$$

Remark 3 Robison [7] derived the sufficient condition (1−1/(N+1))1/d≤c/m<1/d. When remainder(N,d)≠0, Robison's bound is suboptimal unlike Theorem 3. Drane et al. [10] derive a similar result to ours for the case where *d* is odd.

Theorem 4 *Consider an integer divisor* d>0 *and a range of integer numerators* n∈[0,N] *where* N≥d *is an integer. We have that* division(n,d)=division(c⁎n+c,m) *and* remainder(n,d)=division(remainder(c⁎n+c,m)⁎d,m) *for all integer numerators n in the range if and only if*

(14) $$\left(1-\frac{1}{N+1}\right)1/d\leq c/m<1/d.$$

Proposition 2 *Consider an integer divisor* d>0*. We have that d divides* n∈[0,N] *if and only if* remainder(c⁎n+c,m)<c *subject to the condition that*

(15) $$\left(1-\frac{1}{N+1}\right)1/d\leq c/m<1/d.$$

We can check that the conditions of Theorem 3 are met when c=floor(m/d) and m≥d⁎(N−remainder(N,d)+1) as long as *m* is not divisible by *d*. Proposition 2 is satisfied with the more stringent inequality m≥d⁎(N+1).

## 6 Complementarity

When processing numerators and divisors in [0,n], it may be most convenient if the constant *c* is also in [0,n]. In this respect, the multiply-shift and multiply-add-shift results are complementary as first shown by Robison [7]. Suppose that we want to divide all integers n∈[0,N] by *d* in the case where N+1 is a power of two. For example, we may have $N=2^{64}-1$. We want the constant *m* to be a power of two.

When *d* is a power of two, efficient division and remainder routines are available. The quotient requires a single binary shift while the remainder requires selecting the low-weight bits with a mask. Thus suppose that the divisor *d* is not a power of two.

To satisfy the constraints of Theorem 1, Theorem 2, we can pick c=ceiling(m/d) and $m=2^{\mathrm{ceiling}(\log_{2}(d))}⁎(N+1)$. Unfortunately, c=ceiling(m/d) is not in [0,N] which may cause implementation issues. Indeed, if we want to do 64-bit arithmetic on hardware with 64-bit machine words, it is most convenient if all constants fit in 64-bit words. Thus we may try a smaller constant. The choice $m=2^{\mathrm{floor}(\log_{2}(d))}⁎(N+1)$ is convenient since c=ceiling(m/d) is then an integer ∈[0,N]. Unfortunately, it is not a valid choice for all divisors *d*, as per the requirements of Theorem 1, Theorem 2. For example, if $N+1=2^{32}$ and d=19, picking $m=2^{\mathrm{floor}(\log_{2}(d))}⁎(N+1)=2^{4+32}$, we get c=ceiling(m/d)=3616814566. We have that $N-\mathrm{remainder}(N+1,d)=2^{32}-6$. We can verify that $3616814566\geq 2^{4+32}/19⁎\left(1+\frac{1}{\left(2^{32}-6\right)}\right)\approx 3616814565.89$. Thus the conditions of Theorem 1 are not satisfied.

Thankfully, we can fall back on the multiply-add-shift results. Suppose that setting c=ceiling(m/d) and $m=2^{\mathrm{floor}(\log_{2}(d))}⁎(N+1)$ fails to satisfy the conditions of Theorem 1, then we have that

(16) $$\mathrm{ceiling}(m/d)\geq \left(1+\frac{1}{N-\mathrm{remainder}(N+1,d)}\right)m/d.$$

It may be convenient to simplify this equation further. We can multiply both sides by *d*. We have that d⁎ceiling(m/d)=m+d−remainder(m,d) on the left-hand-side. On the right-hand-side, we have *m* plus some quantity that may not be integer, but we can safely apply the ceiling function since the left-hand-side is an integer. After subtracting *m* from both sides, we get

(17) $$d-\mathrm{remainder}(m,d)\geq \mathrm{ceiling}\;\left(\frac{m}{N-\mathrm{remainder}(N+1,d)}\right).$$

(E.g., with d=19 and $N+1=2^{32}$, we get 18≥17.) We want to show that the conditions of Theorem 3 are satisfied when keeping $m=2^{\mathrm{floor}(\log_{2}(d))}⁎(N+1)$ and setting c=floor(m/d). That is, if Theorem 1 fails us, we can use Theorem 3 so that it is always possible to pick c∈[0,N].

We have that $2^{\mathrm{floor}(\log_{2}(d))}>d/2$ and hence m>d⁎(N+1)/2 and therefore d/m<2/(N+1). We assume that *d* is not a power of two. Since we assume N+1 and hence *m* are powers of two, we have that ceiling(m/d)=floor(m/d)+1 and thus from (16)

(18) $$\mathrm{floor}(m/d)+1\geq \left(1+\frac{1}{N-\mathrm{remainder}(N+1,d)}\right)m/d.$$

We can divide by *m* and subtract 1/m to get

(19) $$\mathrm{floor}(m/d)/m\geq \left(1+\frac{1}{N-\mathrm{remainder}(N+1,d)}\right)1/d-1/m$$

(20) $$=\left(1+\frac{1}{N-\mathrm{remainder}(N+1,d)}-\frac{d}{m}\right)1/d$$

(21) $$\geq \left(1+\frac{1}{N+1}-\frac{d}{m}\right)1/d$$

(22) $$>\left(1+\frac{1}{N+1}-\frac{2}{N+1}\right)1/d$$

(23) $$=\left(1-\frac{1}{N+1}\right)1/d$$

(24) $$\geq \left(1-\frac{1}{N-\mathrm{remainder}(N+1,d)+1}\right)1/d.$$

We have shown Proposition 3, which tells us that it is always possible to compute the quotient using c∈[0,N],^2^ selecting the approach using Equation (17).

Proposition 3 *Consider an integer divisor* d>0 *that is not a power of two. Let N be an integer such that* N+1 *is a power of two, then we can compute the quotient of any integer* n∈[0,N] *by d using a constant* c∈[0,N] *as follows. Let* $m=2^{\mathrm{floor}(\log_{2}(d))}⁎(N+1)$*.*

• *if* d−remainder(m,d)<ceiling(m/(N−remainder(N+1,d)))*, we let* c=ceiling(m/d) *and we have* division(n,d)=division(c⁎n,m)*.*
• *Otherwise we let* c=floor(m/d) *and we have* division(n,d)=division(c⁎n+c,m)*.*

The same complementarity exists between the novel theorems for the computation of the quotient and remainder: Theorem 2, Theorem 4. Indeed, if we choose $m=2^{\mathrm{floor}(\log_{2}(d))}⁎(N+1)$ and c=ceiling(m/d), but the conditions of Theorem 2 are not met, then we have that

(25) $$\mathrm{ceiling}(m/d)\geq \left(1+\frac{1}{N}\right)m/d.$$

We have that ceiling(m/d)=floor(m/d)+1 since *m* is not divisible by *d* and thus

(26) $$\mathrm{floor}(m/d)\geq \left(1+\frac{1}{N}\right)m/d-1$$

(27) $$=\left(1+\frac{1}{N}-\frac{d}{m}\right)1/d$$

(28) $$>\left(1+\frac{1}{N+1}-\frac{d}{m}\right)1/d$$

(29) $$>\left(1+\frac{1}{N+1}-\frac{2}{N+1}\right)1/d$$

(30) $$=\left(1-\frac{1}{N+1}\right)1/d.$$

Thus, again, it is always possible to pick c∈[0,N]: if not with Theorem 2, then with Theorem 4, using the analog of Equation (17) to select the correct approach. We formalize the result with the novel Proposition 4.

Proposition 4 *Consider an integer divisor* d>0 *that is not a power of two. Let N be an integer such that* N+1 *is a power of two, then we can compute the quotient and the remainder of any integer* n∈[0,N] *by d using a constant* c∈[0,N] *as follows. Let* $m=2^{\mathrm{floor}(\log_{2}(d))}⁎(N+1)$*.*

• *if* d−remainder(m,d)<ceiling(m/N)*, we let* c=ceiling(m/d) *and we have* division(n,d)=division(c⁎n,m) *and* remainder(n,d)=division(remainder(c⁎n,m)⁎d,m)*.*
• *Otherwise we let* c=floor(m/d) *and we have* division(n,d)=division(c⁎n+c,m) *and* remainder(n,d)=division(remainder(c⁎n+c,m)⁎d,m)*.*

*Ideal divisors*  When N+1 is a power of two, we have shown that it is always possible to pick $m=2^{\mathrm{floor}(\log_{2}(d))}⁎(N+1)$. However, for some divisors, we can pick an even smaller *m*, even with the constraint that *m* be a power of two. Suppose that you would like to compute both the remainder and the quotient, as in Theorem 2. Picking m=N+1 would be especially convenient. (In architectures where the product is stored in a pair of registers, division(x,m) and remainder(x,m) are essentially free if $m=2^{W}$, where *W* is the architecture's word size.) We choose m=(N+1) and c=ceiling((N+1)/d). To satisfy the conditions of Theorem 2, we need that c⁎d<(1+1/N)⁎(N+1)=N+2+1/N. We have that ceiling((N+1)/d)⁎d=(N+1)+d−remainder(N+1,d). Assuming that *d* does not divide N+1, the inequality holds if and only if d−remainder(N+1,d)=1 which is true if and only if *d* divides N+2. We refer to any such divisors as being *ideal*, as they enable us to pick m=N+1. See Table 2.^3^

**Table 2:** Some ideal divisors.

Range [0,*N* + 1)	ideal divisor
[0,2^32^)	641
[0,2^32^)	6700417
[0,2^64^)	274177
[0,2^64^)	67280421310721

We can show that we cannot pick *m* to be a smaller power of two—unless *d* divides N+1. Indeed, if we pick m=(N+1)/2, then the conditions of Theorem 2 require that c⁎d<(1+1/N)⁎(N+1)/2 but 1/N⁎(N+1)/2<1 for N>1 and since c⁎d is an integer, we then must have that c⁎d≤(N+1)/2. Yet we have ceiling((N+1)/(2⁎d))⁎d>(N+1)/d when *d* does not divide N+1.

## 7 Rounding

Instead of computing the integer division (floor(n/d)), we sometimes wish to round the result to the nearest integer. Unsurprisingly and maybe obviously, it is possible to do with an expression of the form floor(z/d) for some integer *z* that depends on *n* and *d*. Hence, our efficient integer quotient computations extend to the computation of the rounded division. Indeed, we have that floor((n+floor(d/2))/d) is the round-to-nearest function; when *d* is odd and *n* is between two multiple of *d*, it rounds up. To round down, we can use floor((n+ceiling(d/2)−1)/d) instead.^4^

It is also possible to handle more complicated scenarios. For example, what if we wish to round to the nearest integer, rounding to the nearest even integer when we are in-between two integers? It is only relevant when the division *d* is even. Let z=n+floor(d/2). Whenever *z* is a multiple of *d* and floor(z/d) is odd, we return floor(z/d)−1, else we return floor(z/d). We can check that an integer is a multiple of *d* efficiently with Proposition 1 for example. The division and the divisibility test can reuse the same intermediate computations.

## 8 Conclusion

Our work shows that a unified approach, with the same precomputed constants, allows the computation of the quotient and remainder, while further providing fast divisibility checks. Thus, for example, an algorithm could check efficiently whether an integer is divisible by another and, in the negative case, compute the remainder while reusing the prior work.

Future work might address the problem of computing generalized expressions such as floor(n⁎x) for integer values *n* and real numbers *x*. For example, we have that $\mathrm{floor}(\log_{2}(5^{x}))=\mathrm{floor}(x⁎\log_{2}(5))$ can be computed as multiplication and a division by a power of two: $(152170⁎x)÷2^{16}$ for x∈(−400,350). We find such optimizations hand-coded in highly optimized algorithms [19]: it might prove useful to formalize the derivation of such routines.

## Declarations

### Author contribution statement

D. Lemire: Conceived and designed the analysis; Wrote the paper.

C. Bartlett, O. Kaser: Conceived and designed the analysis.

### Declaration of interests statement

The first author was supported by the 10.13039/501100000038Natural Sciences and Engineering Research Council of Canada under grant number RGPIN-2017-0391.

### Data availability statement

No data was used for the research described in the article.

### Funding statement

The authors declare no conflict of interest.

### Additional information

No additional information is available for this paper.

## Footnotes

2 This result is not novel [7].

3 They are related to Fermat numbers [18].

4 We define ceiling(x) as the smallest integer that is no smaller than *x*.

## Footnotes

1 Drane et al. [10] have a related bound, but they also have additional constraints on the divisor *d*.
